# Comparison of children with and without dyslexia using functional head impulse test and pediatric balance scale

**DOI:** 10.3389/fneur.2023.1153650

**Published:** 2023-06-02

**Authors:** Gül Ölçek, İlayda Çelik, Yuşa Başoǧlu, Sude Kaymakçı, Emre Gürlek

**Affiliations:** ^1^Faculty of Health Sciences, Department of Audiology, Istanbul Medipol University, Istanbul, Türkiye; ^2^Graduate School of Health Sciences, Department of Speech and Language Therapy, Istanbul Medipol University, Istanbul, Türkiye; ^3^Graduate School of Health Sciences, Department of Audiology, Istanbul Medipol University, Istanbul, Türkiye

**Keywords:** dyslexia, vestibulo-ocular reflex (VOR), vestibular system, functional head impulse test (f-HIT), pediatric balance scale (PBS)

## Abstract

**Purpose:**

The aim of this study is to compare functionality of vestibulo-ocular reflex (VOR) responses to evaluate the functional properties of the vestibular system and daily balance performance in children with dyslexia and children with normal development.

**Method:**

Fifteen participants diagnosed with dyslexia were included in the study group (SG), and 15 healthy participants were included in the control group (CG). All groups underwent Functional Head Impulse Test (f-HIT) and Pediatric Balance Scale (PBS). f-HIT was performed with at least 15 head impulses at 4000-5000-6000°/s^2^ randomly to the right and left in the plane of the horizontal semicircular canal (SCC). Statistical analysis was performed using descriptive statistics and the Mann-Whitney U test.

**Results:**

SG percentage values were obtained lower than CG percentage values. Comparisons between the two groups showed that there was a significant difference in all parameters (4000-5000-6000°/s^2^ and total) in the right-side stimulation, there was significant difference for 4,000 s^2^ and total correct answers in the left side. In addition, although there was no significant difference between the groups in terms of the PBS score, the SG scores were lower (*p* = 0.062).

**Conclusions:**

As a novel test, f-HIT, revealed the difference in functionality of vestibular performance in the dyslexia group. In the dyslexia group, f-HIT may be helpful in evaluating and monitoring the vestibular system.

## 1. Introduction

Specific learning disorder (SLD) is a neurodevelopmental disorder characterized by persistent difficulties in many areas, such as reading, writing, mathematics, listening, speaking, and reasoning, thus poor performance in learning and academic skills for the expected age (1, 2). SLD can be characterized by impaired academic skills despite intact vision, intact hearing, chronological age-appropriate intelligence and education (1, 3). SLD can be subdivided into dyslexia (difficulty in reading and language), dyscalculia (difficulty in mathematics) and dysgraphia (difficulty in writing) according to the difficulty experienced by the person.

The most prominent theory about origin of dyslexia proposes that cognitive deficiencies in phonological processing, which can be defined as ability to decode and manipulate phonemes or difficulty associating letters with the appropriate sounds (4–7). In addition to phonological and literacy difficulties in dyslexia, many studies have focused on the effects of the cerebellum and its functions (8–10). The cerebellum could contribute to reading in various aspects, including eye movements, language and spatial processing, working memory, and skill acquisition and automaticity (8). Thus, while cerebellar dysfunction is not likely the primary cause of dyslexia, impairments on cerebellar tasks—including eye movement control, postural stability, and implicit motor learning could be shown in children and adults with dyslexia (9). Moreover, vestibulo-cerebellar dysfunction can be addressed in the dyslexia group because the cerebellum is inextricably linked to the vestibular system (11, 12). Therefore, altered vestibular function may exist in this group, which may contribute to balance and gait disturbances (13).

It has been reported that children with dyslexia have worse postural performance than children with normal development (14–16). Poor postural performance in children with dyslexia may be due to impairment in visual, proprioceptive, and vestibular systems or to the integration of these sensory cues that contribute to appropriate motor activity (16, 17). For this reason, it is important to evaluate posture, gait, general motor skills, and balance skills in children with dyslexia.

The vestibular system produces reflexes that are crucial for stabilizing the visual axis (gaze) and maintaining head and body posture (18). When the head rotates with angular velocity in the horizontal and vertical (anterior and posterior SCCs) planes, eye movement occurs in the opposite direction to that of the head (19). This reflex, called the VOR, stabilizes the image on the retina during rapid head movements and can be measured utilizing the vestibular testing approaches such as the head impulse test (HIT) (19). The fixation of an image on the fovea is mainly based on the activity of the vestibular and visual systems (20). The activity of each of these systems varies according to the frequency of head movement. For example, at low frequencies (< 0.1 Hz), the visual system is dominant; at mid frequencies, the vestibular and visual systems interact together to stabilize the gaze; at high frequencies (between 1 and 5 Hz), only the vestibular system is activated (21).

Halmagyi and Curthoys described rapid horizontal head rotation, Head Impulse Test (HIT), to use as an important clinical screening (22). The use of this test has increased with the development of a high-speed video HIT system (vHIT). vHIT is easy to use as a clinical tool and has been validated as a diagnostic test of semicircular canal function (23). While the vHIT provides a gain value that summarizes the behavior of the VOR, another similar test, f-HIT, provides data on the ability to recognize the orientation of a Landolt C optotype (24). While vHIT does not provide direct information on the functional effectiveness of the motor response, f-HIT is a functional measure of the VOR as it measures the ability to read and maintain clear vision during head movement (24). Thus, f-HIT can provide information about the vestibular system by providing a functional measurement of the VOR. In the test, the individual is asked to identify an optotype presented briefly during passive head impulses in each SCC plane, and the percentage of correct responses is recorded.

The aim of this study was to compare functional VOR responses and balance skills using f-HIT and PBS in children with dyslexia and children with normal development. In the current study, two specific hypotheses were tested:

i. Children with dyslexia will show worse results in terms of static and dynamic balance in PBS than children with normal development.
ii. Children with dyslexia will show worse results in terms of functional VOR in f-HIT than children with normal development.

## 2. Method

The study was conducted at a special education and rehabilitation center between March and May 2021. This study was conducted in accordance with the Declaration of Helsinki and approved by the local ethical board (Decision no: 784/2021, Istanbul Medipol University, Non-Invasive Clinical Research Ethics Committee). Written informed consent was obtained from the parents of all the participants.

### 2.1. Subjects

G^*^Power 3.1 program was used to determine the study sample size. Type 1 error (alpha) was 0.05, Type 2 error (β) was 0.20 (power = 0.80), and effect size was 0.8. It was determined that there should be a minimum of 26 participants in each group for the study. However, due to our limited access and excluded participants during the COVID-19 pandemic at that time, 15 participants were included in each group.

Children with a diagnosis of SLD were included in the SG. Children with a SLD diagnosis have a “Special Needs Report For Children,” which is diagnosed as “Mixed disorder of scholastic skills” by the health board, which includes physicians from six different branches. Children with SLD were evaluated in the Guidance and Research Center after the health committee report and were diagnosed with SLD. In addition to these reports, 15 participants with “dyslexia” symptoms for at least 6 months and who received special training for “dyslexia” for at least 6 months were included in the SG. The speech and language therapist checked whether the children met the necessary criteria, related documents, special education processes, and dyslexia symptoms.

SG inclusion criteria were: (1) have been diagnosed with a “specific learning disorder (dyslexia)”, (2) ability to adapt to the test, (3) not having any obstacle to restrict the neck movement, (4) not using any assistive devices to assist walking. Fifteen healthy participants were included in the control CG. CG inclusion criteria were: (1) not having cognitive or mental problems, (2) being able to adapt to the test, (3) not having an obstacle to restrict the neck movement, and (4) not using any assistive devices to assist walking.

Whether the participants had a normal vestibular system and hearing system or not was confirmed by the anamnesis (clinical history) taken from their families. Questions about gender, having a middle ear infection in the last 3 months, having hearing loss, having tinnitus or buzzing, having dizziness, visual problems, migraine symptom or diagnosis, BPPV diagnosis and history, head trauma diagnosis and history, systemic disease diagnosis, and neurological disease diagnosis were asked. In the questions, diagnostic criteria consensus document of the committee for the classification of vestibular disorders of the Bárány Society and the International Headache Society (25).

### 2.2. Procedure

f-HIT and PBS were administered to all the groups.

#### 2.2.1. Functional head impulse test

**Figure 1:**
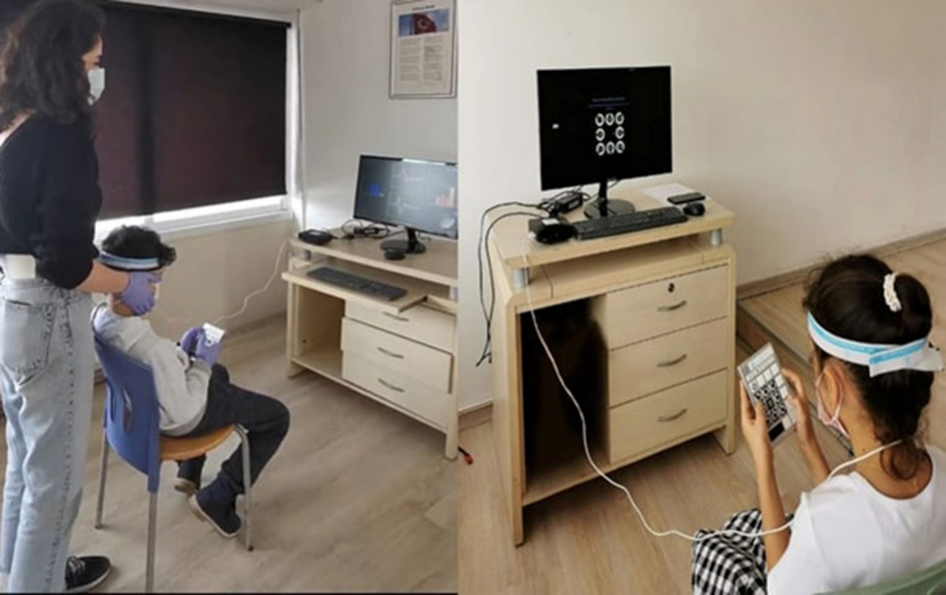
Set up of the f-HIT performed in a special education and rehabilitation center.

The procedure was performed using an f-HIT system (Beon Solutions, Zero Branco, Italy). The participants were positioned 1.5 m away from the f-HIT computer monitor (Figure 1). During testing, a gyroscope was mounted on the children's heads to test their VOR and to measure their head angular velocity.

The same person applied the test to all participants. First, static visual acuity (SVA) was evaluated prior to the procedure. While the size of the Landolt C optotype was 1.0 LogMaR, the optotype size decreased after every 3 correct answers given by the participant. Thus, the appropriate minimum threshold value was found for each participant. The size of the optotype was adjusted for every subject separately and remained constant during testing.

A minimum of 10 passive head impulses were administered varying in acceleration from 4,000 to 6,000°/s2' in each direction in the horizontal plane. The participant looked at the Landolt's C optotype in one of eight possible orientations. An optotype was presented on the computer screen randomly for 80 ms. Afterwards, while their head was turned, the participants had to recognize the optotype and choose the correct C optotype. The absolute outcome was the percentage of correct answers (%CA) for each side, as calculated by the fHIT system.

#### 2.2.2. Pediatric balance scale

PBS, a version of the Berg Balance Scale for children, is used to evaluate functional balance of children in activities of daily living (26). Pediatric version of Berg's balance scale is used to evaluate static and dynamic balance in children aged 3–15 years with mild-to-moderate motor impairment (26).

The scale comprises 14 items including sitting balance, standing balance, sitting to standing/standing to sitting, transfers, stepping, reaching forward with outstretched arm, retrieving object from floor, turning, and placing foot on stool items. Each item is scored from 0 points (lowest function) to 4 points (highest function) with a maximum score of 56 points.

### 2.3. Data analysis

Descriptive statistics were used, including mean values, standard deviations, frequency and percentage. The Mann Whitney U test used to compare paired groups (4,000°/s^2^, 5,000°/s^2^, 6,000°/s^2^). All analyses were performed using the IBM SPSS 22.0 version program. The statistical significance level was set as *p* < 0.05. Statistical significance level was determined as *p* < 0.05.

## 3. Results

The mean age of the SG was 9.80 ± 1.99 years (range 6–14), and there were six female and nine male participants; the mean age of the CG was 9.13 ± 2.42 years (range 6–12) and there were seven female and eight male participants. Table 1 shows descriptive statistics results.

**Table 1:** Socio-demographic information of the participants.

**Group**	***N***	**Age** **mean ±SD**	**Min-Max**	***P*-value** **(Mann Whitney-U)**
Control group	15	9.13 ± 1.99	6–12	0.418
Study group	15	9.80 ± 2.42	6–14	

There was no significant difference between the CG and the SG in terms of mean age (*p* = 0.418). Moreover, for all participants, there was no significant difference in mean age between female participants and male participants (*p* = 0.879). A homogeneous distribution was obtained in terms of age, according to groups and gender.

The mean SVA of SG is 0.46 ± 0.23 (range 0.1–1), while the SVA of CG is 0.34 ± 0.24 (range 0.1–1). There was no statistically significant difference between the two groups (*p* = 0.066) (Table 2).

**Table 2:** F-HIT and PBS results of study and control groups.

**Parameter**	**Control group (Mean** ±**SD) (Min-Max)**		**Study group (Mean** ±**SD) (Min-Max)**		***P*-values (Mann Whitney U Test)**
Static visual acuity	0.34 ± 0.24	0.1–1	0.46 ± 0.23	0.1–1	0.066
Left 4,000°/*s*^2^	96.66 ± 5.99	83–100	84.33 ± 12.97	56–100	0.004^*^
Left 5,000°/^2^	93.73 ± 9.42	75–100	87.06 ± 10.47	69–100	0.080
Left 6,000°/*s*^2^	97.20 ± 7.54	75–100	89.93 ± 15.35	50–100	0.098
Left total	95.86 ± 6.72	79.30–100	87.07 ± 9.22	66.6–97.33	0.003^*^
Right 4,000°/^2^	96.46 ± 9.64	67–100	85.66 ± 16.67	50–100	0.032^*^
Right 5,000°/*s*^2^	94.86 ± 11.13	60–100	85.73 ± 12.72	54–100	0.016^*^
Right 6,000°/*s*^2^	90.53 ± 12.58	67–100	77.73 ± 17.87	48–100	0.006^*^
Right total	93.93 ± 6.58	82–100	83.68 ± 11.15	61.30–100	0.006^*^
PBS	51.73 ± 1.43	50–55	50.20 ± 2.54	45–54	0.062

^*^p<*0.05*.

**Figure 2:**
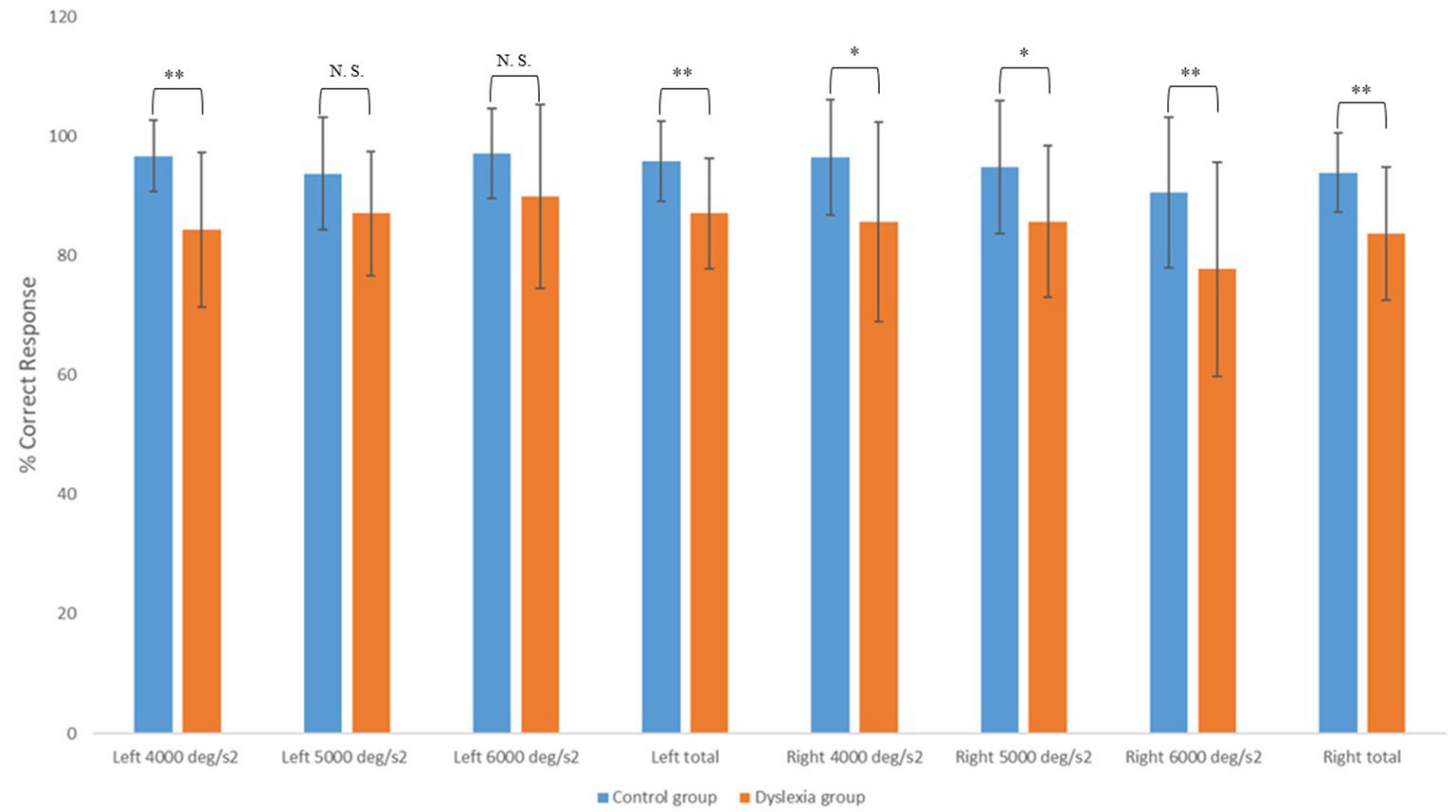
Bar Graphic of f-HIT results of SG and CG. *SG, Study Group; CG, Control Group. *N.S., not significant. *p < 0.05, **p < 0.01.

The values of the participants' f-HIT and PBS parameters were shown in Table 2. The bar graph of the detailed f-HIT results of the groups is given in Figure 2. According to the comparison between the SG and CG, left 4,000°/s^2^, right 4,000°/s^2^, left total and right total values were found to be statistically significant (Table 2). On the other hand, no significant difference was observed between the groups in the left 5,000°/s^2^ and left 6,000°/s^2^ (Table 2). In addition, although there was no significant difference between the groups according to the PBS score, the control group's scores were higher (*p* > 0.05, *p* = 0.062).

## 4. Discussion

This study aimed to compare the f-HIT and PBS results of children with dyslexia and those with normal development. According to the f-HIT result, functional performance of the VOR was close to perfection in the healthy children tested, granting them clear vision during head motion. As we questioned in our study, we observed that children with dyslexia had significantly poor performance on the f-HIT. Interestingly, there was a significant decrease in the rate of correct answers in the SG compared to the CG, especially on the right side. Head acceleration of the SG to the right affected the optotype recognition performance more negatively.

Some studies have reported poor gaze stabilization in children with dyslexia. Jainta and Kapoula examined saccades and vergence control during real-text reading (27). In this study, disparities in fixation were especially noted in relation to poor oculomotor adjustments in children with dyslexia (27). Similarly, findings from the study by Eden et al. showed that dyslexic children had significantly poorer performance in eye movement stability during fixation of small targets, vergence amplitudes, fixation instability than normal children (28). Since f-HIT measures visual fixation and reading ability during head movement, we used f-HIT in our study and it was found that the correct response percentage of the recognized/read optotype during active head movement was lower in children with dyslexia than in children with normal development which is consistent with studies conducted.

Caldani et al. evaluated functional VOR mechanism in children with normal development and children with neurodevelopmental disorders including reading impairment utilizing the f-HIT (29). Children with reading impairment had significantly fewer correct answers than children with typical development (29). In line with the study of Caldani et al., a significant difference was obtained between the CG and SG in our study. Pernet et al., in their study investigating the brain regions that most distinguish children with dyslexia from the normally developing group, they found low gray matter volume in the right cerebellum in the dyslexic group. In our study, poor performance in f-HIT, especially in right acceleration of the head in SG compared to CG is thought to be a potential factor related to the findings of Pernet et al. (30).

Because of the PBS is one of the standardized protocols distinguish between children with normal development and children with mild motor impairments in terms of static and dynamic balance, we used PBS in our study. The Turkish version of the PBS, which validity and reliability studies were conducted, was used in our study (31). However, since there are very limited studies on PBS in children with dyslexia in the literature, we have included articles that make measurements similar to the mechanism of PBS (especially items 4, 6, 7, 8, 9) and examine children with dyslexia in the discussion. Barela et al. measured amplitudes of visual fixation during upright posture and oscillation in an unfixed environment in 12 children with dyslexia and 10 non-dyslexic children (32). They found that dyslexic group had greater oscillation amplitude under all conditions than the non-dyslexic group (32). Pozzo et al. evaluated static posture control performance of 50 dyslexic and 42 normal 10–13 year old children in eyes open and closed positions (33). The center of pressure (CP) displacements along anteroposterior and lateral axes showed a significant difference between the two groups. Dyslexic children showed, on average, more instability in postural parameters with or without vision, with varying and average power frequency. In our study, although no significant difference was found between the PBS scores of the dyslexic group and the control group, the PBS scores of the dyslexic group were found lower (*p* = 0.062).

Many studies in literature on relationship between vestibular dysfunction and cognitive performance have revealed the effect of the vestibular system on cognition (34). It has been shown that the vestibular system has an effect and relationship on visuospatial ability, attention, cognitive processing ability, memory and executive function (34). This evidence shows the importance of the evaluation and intervention of the vestibular system for the development of cognitive skills, especially in groups with neurodevelopmental deficit in many developmental areas such as dyslexia. In our study, we evaluated functional VOR and subjectively static and dynamic balance in children with dyslexia based on the evidence in the literature. We obtained poor functional VOR performance findings in children with dyslexia. Vestibular rehabilitation including functional VOR exercises can be planned to support cognitive development in this population.

## 5. Conclusions

In conclusion, posture, gait and general motor skills were not significantly affected in SG. However, the study revealed that children with dyslexia underperformed significantly on the f-HIT with a low number of correct answers. A new test, f-HIT, revealed the difference in functional vestibular performance in the SG. In patients with suspected or diagnosed dyslexia, f-HIT can be helpful in the evaluation and monitoring of the vestibular system. Considering the role of the vestibular system in neurodevelopmental disorders, such as dyslexia, and its connections with cognition, it is important to evaluate this group using a comprehensive vestibular test battery. The presence of vestibular system deficits in SG can be supported by cross-checking with other tests that evaluate the vestibular system. In future studies, f-HIT findings can be examined in all semicircular canals in a larger sample.

## 6. Limitations of the study

Some limitations are pointed out in this study. The auditory evaluation (pure tone audiometry, acoustic immitansmetry, etc.) and the comprehensive vestibular system evaluation (Videonystagmography, VEMP, etc.) of the participants were not performed with audiological tests. Especially objective tests can be applied in future studies, in addition to participants' self-report. Additionally, there are very limited articles in the literature on PBS applied to children with SLD. This situation has led to limitations in comparing our findings on PBS with other studies. In future studies, different tools can be applied. Another limitation of our study is the small number of participants due to our limited access during the COVID-19 pandemic, so the number of participants may be higher in future studies.

## 7. Presentation(s) or awards at a meeting

This research was presented as an oral presentation on 04.06.2022 at 3rd Oto-Audiology Congress organized by Istanbul Medipol University and won the best paper award.

## Data availability statement

The raw data supporting the conclusions of this article will be made available by the authors, without undue reservation.

## Ethics statement

The studies involving human participants were reviewed and approved by Istanbul Medipol University Non-Invasive Clinical Research Ethics Committee. Written informed consent to participate in this study was provided by the participants' legal guardian/next of kin.

## Author contributions

GÖ, İÇ, and YB contributed to conception and design of the study. İÇ organized the database. YB performed the statistical analysis. GÖ wrote the first draft of the manuscript. GÖ, İÇ, YB, SK, and EG wrote sections of the manuscript. All authors contributed to the data collection process and manuscript revision, read, and approved the submitted version.
